# Is Physical Activity an Efficient Strategy to Control the Adverse Effects of Persistent Organic Pollutants in the Context of Obesity? A Narrative Review

**DOI:** 10.3390/ijms25020883

**Published:** 2024-01-10

**Authors:** Quentin A. Serrano, Sébastien Le Garf, Vincent Martin, Serge S. Colson, Nicolas Chevalier

**Affiliations:** 1 Université Côte d’Azur, LAMHESS, France; quentin.serrano@univ-cotedazur.fr; 2 Université Côte d’Azur, CHU, INSERM, C3M, France; legarf.s@chu-nice.fr (S.L.G.); chevalier.n@chu-nice.fr (N.C.); 3 Université Clermont Auvergne, AME2P, F-63000 Clermont-Ferrand, France; vincent.martin@uca.fr; 4 Institut Universitaire de France (IUF), 75005 Paris, France

**Keywords:** endocrine disruptors, inflammation, insulin resistance, lipid, adipogenesis, microbiota, bariatric surgery, adipose tissue

## Abstract

Obesity affects nearly 660 million adults worldwide and is known for its many comorbidities. Although the phenomenon of obesity is not fully understood, science regularly reveals new determinants of this pathology. Among them, persistent organic pollutants (POPs) have been recently highlighted. Mainly lipophilic, POPs are normally stored in adipose tissue and can lead to adverse metabolic effects when released into the bloodstream. The main objective of this narrative review is to discuss the different pathways by which physical activity may counteract POPs’ adverse effects. The research that we carried out seems to indicate that physical activity could positively influence several pathways negatively influenced by POPs, such as insulin resistance, inflammation, lipid accumulation, adipogenesis, and gut microbiota dysbiosis, that are associated with the development of obesity. This review also indicates how, through the controlled mobilization of POPs, physical activity could be a valuable approach to reduce the concentration of POPs in the bloodstream. These findings suggest that physical activity should be used to counteract the adverse effects of POPs. However, future studies should accurately assess its impact in specific situations such as bariatric surgery, where weight loss promotes POPs’ blood release.

## 1. Introduction

Obesity is an excessive accumulation of fat mass within the body contributing to ectopic lipid deposits. Obesity is clinically characterized by a BMI (Body Mass Index) greater than 30 kg/m^2^ and is linked to metabolic disorders, psychosocial consequences, and impaired quality of life. These deposits depend on the cellular modification of the adipose tissue related to morbi-mortalities. Globally, obesity is increasing and is considered a true pandemic. Its prevalence tripled between 1975 and 2016. Worldwide, more than 660 million adults are obese. Moreover, this increase also affects children and is observed in all socio-economic groups. The latest WHO report specific to the European Region counted 59% of adults and nearly one child out of three (29% of boys and 27% of girls) as being overweight or living with obesity. Android obesity, which is specifically characterized by visceral fat accumulation, may be accompanied by comorbidities such as cardiovascular disease [1], hormone-dependent cancers (National Cancer Institute, Obesity and Cancer 2017), type 2 diabetes (T2DM) [2], and metabolic dysfunction-associated fatty liver disease (MAFLD) [3]. Owing to these comorbidities, obesity is estimated to cause millions of deaths [4] each year worldwide (WHO, 2021). It is therefore crucial to address the causes contributing to the increasing prevalence of obesity. In most cases, no single determinant is exclusively responsible for the development of obesity. In this narrative review, we focus on one of the factors contributing to this multifactorial pathology. Specifically, we highlight the role of exposure to chemical substances, in particular persistent organic pollutants (POPs), in the development of obesity. The impact of POPs on human health has been studied for many years. Their presence has been linked to the development of numerous pathologies. Many studies have demonstrated the effects of POPs on the development of obesity [5,6]. However, the exact mechanisms by which POPs facilitate the development of obesity are still under investigation. Several interesting clues have been identified. Many of these clues may be influenced by physical activity (PA). The use of PA to counteract and prevent pathologies has gained popularity in recent years. In particular, PA is recognized as an easy-to-implement, low-cost intervention with significant protective results for various pathologies. This review will address how PA, through several mechanisms, could help controlling the adverse effects of POPs. The methodology used to select the articles included in the review can be found in Supplementary Materials, Figure S1.

## 2. Persistent Organic Pollutants (POPs)

### 2.1. What Are Persistent Organic Pollutants (POPs)?

POPs are organic compounds coming mainly from pesticides and industrial chemicals. These molecules have four specific properties. Firstly, these molecules have the capacity to be transported over very long distances (e.g., between two countries, traveling through air, water, soil, and the food chain). Secondly, they are bioaccumulable, implying that these molecules enter the organism; have the capacity to accumulate in the organism; and, even more seriously, can be transmitted from generation to generation. Thirdly, they are toxic, which means that they are a significant danger to the health of human beings and to wildlife. Fourthly, they are persistent, meaning that it is difficult to degrade these molecules, and they can stay in the environment for many years (Stockholm Convention, UN environment program).

### 2.2. Dose-Response Relationship and Synergistic Effects of POPs

In many cases, POPs do not follow a linear dose-response relationship [7]. In other words, POPs can be more dangerous at low doses than at moderate or high doses. Some POPs have been reported to have an inverted U-shaped dose-response relationship. While many studies have evaluated the individual effects of several POPs, the human body is affected by mixtures of POPs [7]. The assessment of mixtures is essential, as POPs can have synergistic effects on each other. When a POP is added to a mixture, it can have an additional, multiplying, or antagonistic effect. The principle of antagonism can therefore explain why in certain situations, POPs do not have the expected effect. For example, a POP promoting the development of adipose tissue associated with a POP limiting the development of adipose tissue should theoretically lead to a cancellation of their respective effects. However, it is possible to assume that in case of non-equal concentration of the two POPs and/or different dose response relationships, the results could differ.

### 2.3. POPs Nowadays

Many chemical molecules are no longer authorized on the market because of their POPs-related properties. However, problems remain due to POPs’ relatively long life expectancy, their half-life in organic tissues, and their unconscious industrial production around the world. Another problem consists of the presence of POPs used before their embargo and therefore remaining in the living environment, for example, in building materials. Currently, molecules used before their ban are regularly found in our environment. Furthermore, POPs banned in many countries are still observed and/or produced in specific locations/occasions [8,9]. In addition, some POPs are still used in the context of integrated vector management (e.g., dichlorodiphenyltrichloroethane [DDT]). Eradication of POPs remains difficult because of their characteristics and because many molecular alternatives (e.g., industrial, pesticides) still need to be found, knowing that these alternatives must meet multiple criteria to be considered viable (e.g., yield, price, efficacy, safety). POPs’ ability to travel great distances represents a threat for populations. It should be mentioned that new molecules are regularly added to the official comprehensive list of POPs (established under the Stockholm Convention Listing of POPs in the Stockholm Convention; Stockholm Convention Secretariat, http://www.pops.int/TheConvention/ThePOPs/AllPOPs/tabid/2509/Default.aspx, accessed on 12 March 2023). Human concerns regarding these molecules are due to regular exposure via food and water pollution [10]. Following this exposure, and owing to their lipophilic properties, POPs will be stored in adipose tissue and will be able, under certain circumstances, to be released into the bloodstream [11]. The presence of POPs in the human body may cause many disruptions by affecting physiological mechanisms. In this regard, in vitro and in vivo models and epidemiological research have highlighted that POPs could be obesogens [5].

### 2.4. POPs as Obesogens

At the beginning of the 21st century, links between POPs and adipose tissue were highlighted in the scientific literature. Based on several publications over the last 20 years, the idea emerged that POPs may be determinants of obesity. Indeed, some POPs have an obesogenic effect [5,12]. Simply put, obesogens are chemical substances capable of increasing fat storage, promoting fat production, and reducing fat utilization. Obesogenic molecules can be defined as “chemicals that alter homeostatic metabolic setpoints, disrupt appetite controls, perturb lipid homeostasis to promote adipocyte hypertrophy, stimulate adipogenic pathways that enhance adipocyte hyperplasia, or otherwise alter adipocyte differentiation during development” [13]. More precisely, “obesogens are chemicals that elicit increased white adipose tissue mass (WAT) after exposure in vivo […]. Potential obesogens are chemicals that can induce differentiation of adipocytes in vitro but have not yet been demonstrated to increase WAT accumulation in vivo” as defined by Heindel et al. [5]. The same authors explained that obesogens can affect many different tissues and parameters related to metabolism (e.g., tissue development; inflammation; oxidative stress; circadian rhythms; brain metabolic control; and epigenetic, microbiome, and signaling pathways). Several different categories of POPs are currently directly associated with obesity, such as organochlorine pesticides [OCPs] (e.g., found in insecticides designed to fight against malaria), polychlorinated biphenyls [PCBs] (e.g., found in plastics, paints, and electrical components), per- and polyfluoroalkyl substances [PFASs] (e.g., found in cosmetics, in the textile sector, and in phytosanitary products), and polybromodiphenylethers [PBDEs] (e.g., found in the textile and plastic sectors). Obesogenic effects have also been observed for other categories, like dioxins [14]. It should nevertheless be noted that the presence of multiple cofactors makes it difficult to accurately determine the impact of POPs on human obesity.

Taken together, this reflects the harmfulness of POPs to the body and to the consequences of obesity on individuals and society. There is a need to find solutions to effectively address this problem. Many countries are banning the use of these chemicals, but as explained above, some remain in our environment. Several articles have looked at the impact of different types of diet on exposure to POPs [15,16]. To our knowledge, only a few studies have evaluated the effect of PA on POP concentrations and/or adverse effects. Recent reviews [5,6] have examined the molecular pathways underlying the ability of POPs to promote the development of obesity. From these reviews, several crucial insights emerged, such as adipogenesis, lipid accumulation, insulin resistance/alteration of insulin sensitivity, inflammatory function, and gut microbiota dysbiosis. The practice of PA could positively counteract these effects. It may also promote the elimination of POPs. Following a short explanation of the importance of PA in the context of obesity, the interest of PA for each insight will be discussed.

## 3. PA and POPs in the Context of Obesity

### 3.1. PA and Obesity

PA is defined as “any bodily movement produced by skeletal muscle contraction resulting in an increase in energy expenditure over resting energy expenditure” [17]. Adapted physical activity is a form of PA with a preventive and curative aim considering the clinical and motivational state of the person in order to promote bio-psycho-social well-being [18]. Bio-psycho-social well-being could be defined as a positive interaction between the well-being of the body (e.g., functional abilities, functioning of physiological processes), the well-being of the mind (e.g., behavioral and psychic processes) and the well-being of living interactions (e.g., communication, social/environmental interaction). Each of these aspects has its own characteristics. The body, mind, and interactions of living beings are parameters that must be combined to provide the best possible support for an individual. The proven effects of PA on the reduction/prevention of diseases and associated disorders, particularly obesity-related comorbidities, and more globally on the improvement of quality of life, have led to the inclusion of the “physical activity prescription” in the public health codes of many countries.

According to the American College of Sports and Medicine (ACSM, 2013) and EASO consensus statement [19], PA recommendations to prevent obesity are similar to those for the general population (i.e., at least 150 min/week of moderate PA or at least 75 min/week of vigorous PA combined with resistance training at least two times/week). However, to gain more benefits and allow for maintenance of the positive adaptations obtained after weight loss, more than 300 min/week of moderate to vigorous PA is recommended (ACSM, 2011 and 2013). Despite this, it is nowadays considered that any PA, even less than the recommendations previously mentioned, is better than no activity. Nevertheless, specific PA programs have proven more effective that the general recommendations. To be more precise, a meta-analysis highlighted a significantly greater decrease in waist circumference with combined or aerobic training alone than resistance training alone [20]. Moderate- to high-intensity aerobic physical training is the most effective type of PA to significantly reduce visceral adipose tissue (VAT) in obesity [21]. Furthermore, an intervention on the mobilization of WAT, in particular reducing VAT, plays a key role in treating obesity-related inflammation. To conclude the meta-analysis observations, PA promotes the mobilization of free fatty acids (FFAs) from their storage sites by enhancing the sensitivity of adipocytes to the lipolytic influence of catecholamines and their muscular utilization through activation of the AMPK/PGC1α signaling pathway [22].

PA is recognized for its many beneficial effects on obesity. For example, PA can improve physical capacities (e.g., muscle strength) [23], improve cognitive capacities [24], change body composition [25], influence metabolism [25], reduce inflammation [25], and affect the hormonal system [25]. In this article, we highlight how POPs can increase adipose tissue development, increase fat accumulation, reduce insulin sensitivity, increase inflammation, and modify gut microbiota. PA could therefore counteract the obesity-related adverse effects of POPs. PA may also limit the development of obesity by reducing POP blood concentrations. This specific point will also be discussed later in this review.

### 3.2. The Link between POPs, PA, Adipogenesis, and Lipid Accumulation

Adipogenesis is the differentiation process whereby preadipocytes become adipocytes. Adipogenesis favors lipid accumulation and the development of adipose tissues. Adipogenesis and lipid accumulation can be increased through several physiological pathways and transcription factors. Among these, PPARγ, STATs, C/EBPα, C/EBPβ, C/EBPδ, and SREBP-1 are central [26]. PPARγ is a nuclear receptor and a transcription factor regulating adipocyte differentiation and gene expression. STATs are proteins able to increase adipocyte differentiation in cases of ectopic expression. C/EBPs are a set of proteins that have an active role at different phases of adipogenesis. SREBP-1 is another transcription factor and protein implicated in lipogenesis. Interestingly, many of these transcription factors interact with each other during adipogenesis. For example, the ectopic level of C/EBPβ can be related to *PPARγ* expression [27], and this can also be true for the ectopic expression of STAT5 [28].

It is established that some obesogenic POPs have the ability to increase adipogenesis [29]. POPs can influence the activity and/or expression of the main molecular pathways involved in lipid metabolism through transcription factors (i.e., PPARγ, C/EBPα, C/EBPβ, and SREBP-1), as observed under in vitro conditions [30,31,32]. This would lead to an increased differentiation of adipocytes. C/EBPδ may also be an interesting factor to consider when evaluating POPs’ effects, but there is a lack of studies on this subject. Most studies have investigated POPs with antiadipogenic properties. One study did not find any effects of PDBE 99 exposure on *C/EBPδ* [31]. In addition, an increased expression of adipogenesis-specific gene markers such as LPL were observed in in vitro conditions [30].

To our knowledge, there is a lack of evidence about the direct or indirect effects of PA in relation to adipogenesis and lipid accumulation in the presence of POPs. Nevertheless, it is known that PA can be involved in the activity and/or expression level of PPARγ [33,34], C/EBPα, C/EBPβ, and C/EBPδ [35,36] as well as for SREBP-1 [37], as observed in animal models. Thus, better control of adipogenesis through regular PA could promote a reduced risk of obesity development. It is possible to expect that PA, by regulating the expression and activation of the previously cited adipogenesis factors affected by POPs, may prevent the development of obesity. Finally, it is important to mention that, to our knowledge, no study has investigated the direct relationship between PA, the previously cited transcription factors, and POPs.

Glucocorticoid receptor (GR) is a key factor in adipogenesis. A study indicated the inhibition of adipogenesis with GR antagonists [38]. In contrast, another study observed an increase in adipose tissue development factors (e.g., cell proliferation and triglyceride accumulation) with a GR agonist [39]. Interestingly, some POPs may disrupt GR expression [40]. Independently of the effect of POPs, the protective effect of PA on GR has already been established [41]. Although studies seem to indicate that GR and POPs can interact with each other [42], the ability of PA to counteract the adverse effects of POPs via an effect on GR remains to be established. The understanding of this relation would allow for a better comprehension of how PA may contribute to reducing POPs’ adverse effects in the context of obesity.

Other molecular mechanisms are involved in the development of adipose tissues and lipid accumulation. The Notch pathway, TYK-2/STAT-3 pathway, FABP, FAS, AhR, and hormonal actions are also central. The Notch pathway is a signal transduction factor participating in lipid metabolism. The inhibition of the Notch pathway can reduce obesity development during a high-fat diet [43]. TYK-2 is part of the JAK family and refers to an enzyme as well as a gene encoding the enzyme. It is known that the alteration of the TYK-2/STAT-3 pathway can increase obesity development [44]. TYK-2 and STAT-3 are part of the JAK/STAT signaling pathway. FABP is a fatty acid/lipophilic substances transport protein, and FAS is an enzyme participating in fatty acid biosynthesis. AhR (aryl hydrocarbon receptor) is a ligand-activated transcription factor implicated in adipocyte differentiation and related to PPARγ activity [45]. Its inhibition can prevent the development of obesity [46]. Even more interesting, the inhibition of AhR has the potential to reverse obesity [47]. Key hormonal factors implicated in adipogenesis and lipid accumulation include epinephrine, norepinephrine, estrogens, and androgens. Epinephrine and norepinephrine are hormones highly implicated during lipolysis. Androgens have antiadipogenic effects, and estrogens have proadipogenic effects, following the results observed on in vitro rat preadipocytes [48]. However, the pro and antiadipogenic effects of androgens and estrogens can be debated [26].

POPs have the capacity to negatively alter β-oxidation, to promote lipotoxicity, to alter lipid export, and to promote triglyceride synthesis, contributing to enhanced lipid accumulation in the body [49]. Studies have reported differences between a low-fat diet and high-fat diet. POPs may also be linked to the development of obesity by increasing activation of the Notch pathway, by causing alteration of the TYK-2/STAT-3 pathway, and by increasing FABP expression and FAS upregulation, observed under in vitro conditions [32,50,51]. The ability to limit lipid accumulation is also impaired by POPs. Indeed, POPs are associated with an inhibition of adrenergic-, epinephrine-, and norepinephrine-induced lipolysis pathways [52]. Other studies have reported the alteration of mitochondrial function with high POPs concentrations in vivo [53]. The ability to prevent the thermogenic response of adipocytes with an AhR agonist (i.e., PCB 126) was also observed under in vitro conditions [54]. For these reasons, and because of its importance in energy expenditure, the thermogenic response principle is central in the context of obesity. A recent in vitro study evaluated the effect of a mixture including 29 POPs [55]. Although four of these POPs were AhR agonists, the global mixture was assessed to antagonize AhR activity. However, and according to the results of the same study, some POPs antagonizing AhR activity can have a non-monotone and non-linear dose–response relationship. The obesogenic effect of POPs known to be AhR agonists has been demonstrated in other articles [14]. The alteration of AhR activity promoting the development of obesity could depend on POPs’ mixture composition and individual POP concentrations.

In the context of factors related to lipid accumulation, PA can positively influence some molecular pathways such as the Notch pathway, and studies show that PA can modulate the TYK-2/STAT-3 pathway [56,57]. The levels of proteins involved in fatty acids transport and related to FABP would also be modified. For example a study observed a decrease of FAB4 plasma levels following PA [58]. In addition, a study reported the capacity of chronic PA to reduce FAS activity on obese rats but not for lean rats [59]. Moreover, it has been shown that PA improves mitochondrial respiration and, more globally, mitochondrial function, including protein content [60,61]. Furthermore, PA is known to influence the production of hormones such as adrenaline [62], which could counteract the inhibition of adrenergic-, epinephrine-, and norepinephrine-induced lipolysis caused by POPs. It is important to mention that PA is recognized as a central approach in increasing energy expenditure [63] given that studies demonstrate its impact on the thermogenic function of adipose tissue and on reducing AhR and cytoplasmic levels, notably in humans [64,65]. The positive impact of PA on β-oxidation, lipid export, and triglyceride synthesis is also well known. New research should focus on establishing if these results are still observable in rodent models exposed to different POPs. To our knowledge, no study has assessed the ability of PA to directly counteract the adverse effects of POPs on the Notch pathway, on the TYK-2/STAT-3 pathway, on FABP, on FAS, or on AhR. One study observed that an intervention including diet and PA may attenuate the obesogenic effect of PFASs [66].

A study indicated that POPs could disrupt the translocation and transactivation of androgen receptors [67]. This result should be taken with caution because it could change depending on the POP concentrations and combinations. This study also observed different results between different POPs mixtures or compounds, emphasizing the importance of the cocktail effect. Another study reported a low but significant agonist effect of DDT on estrogenic activity [68]. Several studies assessed the effect of PA alone with these hormones. An increase in dihydrotestosterone (i.e., androgen metabolite) was observed following PA and more precisely resistance training [69]. For estrogens, a meta-analysis concluded that PA reduced estradiol body concentrations but also positively influenced SHBG (sex hormone binding globulin), a hormonal regulator that reduces hormone activity [70]. To our knowledge, no study has directly compared PA and POPs on androgens and estrogens.

### 3.3. The Link between POPs, PA, and Insulin Resistance/Insulin Sensitivity

The reduction of insulin sensitivity is one of the determinants of obesity, and relations between insulin resistance and obesity are well known [71,72]. A lack of insulin sensitivity and insulin resistance are partially caused by the alteration of various physiological factors of insulin regulation. Examples of these factors include JNK, IRS, PTEN, the PI3K-Akt pathway, and GLUT4. JNK and PTEN are modulators of key parameters of insulin sensitivity. In some circumstances, they can participate in insulin resistance. IRS and the PI3K-Akt pathway are two key elements of insulin sensitivity leading to the activation of GLUT4. In return, GLUT4 helps to control glycemia by transporting glucose. To be more precise, JNK is a signal transducer implicated in cellular anabolism and catabolism related to insulin sensitivity, obesity, and insulin resistance [73]. JNK also has a key role in macrophage activity. IRS is a protein, the role of which mostly consists of transmitting intracellular signals coming from insulin receptors. They participate to glucose metabolism. PTEN is a tumor suppressor known for its ability to inhibit the PI3K-Akt pathway [74] and for GLUT4 translocation. Dysregulation of the PI3K-Akt pathway can lead to insulin resistance. Alteration of GLUT4 activity can limit glucose transport. Additionally, inflammatory factors such as TNFα can promote insulin resistance. Finally, it is interesting to note that thyroid function is a master regulator of lipid homeostasis and glycemia homeostasis. Its dysregulation may contribute to insulin resistance development [75].

As explained by a recent review [6], POPs can influence the previously cited mechanisms of insulin sensitivity. Thus, some POPs can increase *TNFα* expression and influence the JNK molecular pathway [76,77]. In addition, POPs can influence thyroid dysfunction, to which ROS and the JNK pathway may contribute [77]. Other insulin resistance factors, such as dysfunction of insulin signaling and negative impacts on the Akt pathway (i.e., reduction of phosphorylated Akt), on GLUT4 expression [78], and on insulin receptor/IRS were evidenced [79]. Moreover, POPs are known to increase PTEN expression [78], which inhibits the PI3K enzyme and Akt pathway [80]. This insulin resistance is even more problematic as it contributes to ROS production through its link with hyperglycemia and ROS, which promote lipid peroxidation. Lipid peroxidation is part of a vicious circle because it in turn promotes insulin resistance. A recent review summarized the major interactions between oxidative stress, inflammation, hyperglycemia, and insulin resistance [81]. In addition, some researchers have proposed that insulin resistance could cause an uncontrolled release of POPs into the bloodstream [82]. POPs are determinants of obesity, which itself is a determinant of the development of insulin resistance. In conclusion, POPs are able to reduce insulin sensitivity by both indirect and direct mechanisms. For example, they may promote inhibition of key insulin-sensitivity factors or disrupt molecular mechanisms.

It is well known that PA is an effective strategy to prevent and reduce insulin resistance. Studies showed an improvement in insulin sensitivity through PI3K/Akt pathway activity, the reduction of JNK activity [83], glucose transport via GLUT4 [84], and insulin receptor tyrosine phosphorylation as well as IRS phosphorylation [85,86]. A study reported many combined potential beneficial effects of PA on specific molecular pathways that impact insulin transduction [87], such as GLUT4 expression, insulin receptor expression, IRS2 protein expression, insulin-stimulated receptor tyrosine phosphorylation, insulin-stimulated tyrosine phosphorylation of IRS1, PI3K activity, and insulin-stimulated Akt phosphorylation. Positive effects on the activity of PTEN have also been observed following PA, and these effects could participate to promote insulin sensitivity [88]. To summarize, unlike POPs, which can reduce insulin sensitivity, PA can improve insulin sensitivity. Directly comparing POPs exposure and PA on insulin resistance is thus essential.

**Figure 1:**
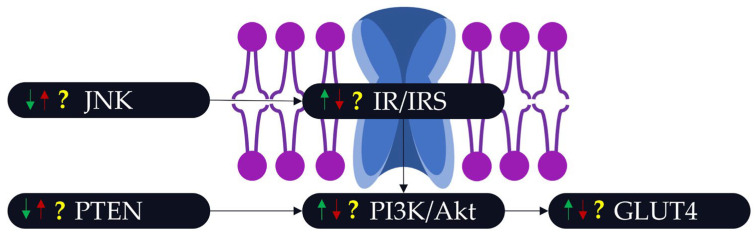
Potential interactions between POPs and PA on insulin sensitivity. Numerous mechanisms interact to ensure stable insulin sensitivity. However, POPs can disrupt these mechanisms (red arrows), and conversely, PA could limit their disruption (green arrows). However, these results are based on studies that did not directly compare exposure to POPs and the practice of PA. There is therefore a lack of knowledge about the level of protection that PA can provide following exposure to POPs (yellow question marks).

Few studies have investigated the beneficial effect of PA as a countermeasure to the adverse effects of POPs on insulin resistance. A study compared the effect of tetrachlorodibenzo-p-dioxin (i.e., a POP) and PA on glucose metabolism and the IRS/PI3K/Akt pathway [89]. The results showed that while PA had positive effects on some parameters (e.g., IRS2), the ability of PA during POP exposure to counteract altered insulin sensitivity appeared to be limited. A second study indicated that children with maternal exposure to PFAS and reporting high PA scores displayed null HOMA-IR indexes in contrast to children with lower PA scores [90]. The interaction between DDT and PA was noted in 1977 during the assessment of blood glucose levels on rats [91]. This study also showed that, when exposed to DDT, PA increased the insulin levels of exercised rats in comparison with sedentary rats. A recent study investigating different PFASs demonstrated that an intervention including diet and PA could protect the individual from diabetogenic effects [92]. Our knowledge about the interaction of POPs and PA and their effect on insulin sensitivity remains limited. Thus, it would be interesting to determine the cumulative effect of PA and POPs on insulin-related factors like JNK, PTEN, and GLUT4. A schematic representation of possible interactions between POPs and PA on insulin sensitivity is shown in Figure 1.

### 3.4. The Link between POPs, PA, and Inflammatory Function

POPs may influence certain factors related to inflammation, which is associated with insulin resistance and obesity [93]. The easiest way to observe the inflammation caused by chemical substances is to measure proinflammatory cytokines (e.g., IL-1β and TNFα), anti-inflammatory cytokines (e.g., IL-10 and IL-4), and inflammatory markers (e.g., C-reactive protein). It is also possible to measure hormone concentrations more or less directly related to inflammation. Thus, leptin and adiponectin should be considered. As a reminder, leptin is recognized for its influence on satiety as well as inflammation. Another molecule, adiponectin, is recognized for its anti-inflammatory capacities, its influence on insulin sensitivity, and its influence on lipid/glucose metabolism.

Intestinal exposure to POPs can promote NF-κB protein activation via the ATM/NEMO pathway, leading to an increase in IL-6 and TNFα, as observed in a rodent model [94]. NF-κB is a protein implicated in cytokine production but also in insulin resistance. POPs are also thought to be linked to inflammation via the AhR protein and expression of the inflammatory cytokine IL-1β, as found in an in vitro experimentation [95]. In addition, this study demonstrated an increase in macrophage polarization and a significant increase in CCL2, CCL3, and CCL4, cytokines involved in the inflammatory function. Other inflammation factors appear to be impacted by POPs exposure. A human study showed that these molecules contribute to altered levels of IFNγ, IL1-β, IL-2, IL-5, IL-8, IL-12p70, IL-17A, TNFα, and TNFβ [96]. Interestingly, another study indicated that POPs could be the cause of a chronic pro-inflammatory state [97]. Inflammatory markers such as the C-reactive protein seem to increase in the presence of some POPs, while others do not show any effect or are inversely associated with it [98]. The impact of POPs on inflammation-related factors may also involve alteration of leptin signaling, leptin gene expression, and leptin receptor expression [99]. This discovery is therefore linked to the phenomenon of leptin resistance, which is known to facilitate the development of obesity. Adiponectin can be negatively associated with POPs [100], as observed in a human study. The link between POPs and adiponectin could be explained indirectly by the hypoxia phenomenon caused by adipose tissue expansion. However, this may not be applicable to all POPs [101]. Other factors promoting inflammation that are impacted by POPs, such as ROS [102,103], adipose tissue dysfunction, altered lipid metabolism, and macrophage infiltration [104], should also be considered. According to the result of a study [105], intestinal inflammation can also be caused by POPs in an AhR-dependent manner.

Regarding factors related to inflammation, PA limits the activation of the NF-κB protein, in particular by promoting the increase of its inhibitor IκB [106]. Another study showed that PA promotes the release of anti-inflammatory cytokines such as IL-1ra and IL-10. This increased concentration of anti-inflammatory cytokines results from the production of IL-6 following PA [107]. Together with this IL-6 production, PA may inhibit other inflammatory factors like IL-1β and TNFα and this can result in changes to hormonal status, PA intensity, and PA duration [107,108]. A meta-analysis focusing on overweight and obese adults indicated that PA in association with caloric restriction can be more efficient than caloric restriction alone to reduce TNFα levels. The results were dependent on lifestyle behaviors (i.e., sedentary levels) [109]. In parallel, we have already mentioned the influence of PA on the AhR protein, the inflammatory role of which was discussed earlier. Although it is recognized that immediate assessment of inflammation following PA elevated inflammatory markers, chronic PA can reduce CRP concentrations [110]. A meta-analysis [25] highlighted that PA was associated with a decrease in leptin and an increase in adiponectin in children. Thus, PA can help to reduce leptin resistance [111]. Interestingly, both hormones are strongly linked to the insulin resistance phenomenon and to inflammatory function [112]. PA is also essential for reducing oxidative stress, and different training modalities may have different effects and targets [113]. Furthermore, oxidative stress is known to be linked to insulin resistance, which was previously described as a major determinant of obesity and POPs’ release into the bloodstream.

All the previously cited articles only assessed the effect of PA on inflammation without POPs exposure. Evidence for comparable effects in organisms contaminated by POPs remains scarce. PA was recognized as a key approach to limit the oxidative stress caused by some POPs by increasing the activity of antioxidant enzymes such as SOD, CAT, GSH-Px, and MDA scavenging [114]. A study reported a decrease in IL-6, CCL2, and macrophages in the PA group exposed to one PCB in comparison with a sedentary group. The results also showed reduced oxidative stress and increases in various antioxidant enzymes (e.g., GPx) [115]. Regarding hormones, a study assessed the effect of PA on leptin–adiponectin ratio following maternal exposure to specific PFASs [90]. Interestingly, this ratio was used to assess adipose tissue dysfunction. Another study reported links between PFASs, leptin, and adiponectin, but PA did not appear to prevent the related alterations [116]. A study also demonstrated the interaction between PA and POPs on inflammatory factors but in the context of wound healing [117]. The study observed that IL-1β levels, TNFα levels, CCL2 levels, and IL-6 levels following PA changed the function of POPs exposure. With the knowledge linking PA, inflammation, and POPs being limited, future studies should explore this research area.

### 3.5. The Link between POPs, PA, and Gut Microbiota

Exposure to specific POPs can cause dysbiosis of the gut microbiome, as observed in a mouse model [118]. A recent review highlighted how dysbiosis can contribute to the development of obesity [119]. Additionally, it is important to mention that the adverse effects of obesogenic chemicals may be facilitated by the alteration of gut microbiota [120]. Another study reported alterations of gut microbiota, metabolism, and inflammatory function when exposed to POPs [105]. The study also reported an important relationship between POPs, gut microbiota, and AhR. Our narrative review already exposed the effect of AhR during obesity development.

A recent review focused on PA and its effect on the gut microbiota. One of the objectives of the review was to determine how different PA could modulate gut microbiota, for example, by affecting the *Bacteroides*–Firmicutes ratio or the gut microbiota diversity [121]. Interestingly, PA in obese children can reduce the abundance of *Proteobacteria* phylum, and *Proteobacteria* are known to be associated with the gut microbiota profile of adult people living with obesity [122,123]. It is possible to suggest that PA may protect individuals from microbiota dysbiosis in the early stages of their life when exposure to POPs is critical.

Following these insights, it was necessary to assess if there was a clear interaction between PA and POPs exposure. One study found that when mice were exposed to PCBs, the abundance of *Proteobacteria* was decreased, but PA attenuated this decrease [118]. A second study presented more mitigated results when observing the effect of PA on the gut microbiota of mice following maternal exposure to PCBs [124].

### 3.6. Effects of PA on the Mobilization of POPs

Some of the above-mentioned studies suggest that PA could be a valuable strategy to counteract POPs’ adverse effects. However, one may also argue that PA could also promote the release of POPs into the bloodstream. This may be associated with adverse effects. Few articles have focused on the effects of PA on POP blood concentration levels. It is therefore essential to assess if PA can increase POPs’ excretion and elimination or, in contrast, increase their interaction with organs.

Following their entrance into the body, most POPs can be found in adipose tissues, where they are stored, but also in the blood flow. As suggested in a study [125], POPs moving in the bloodstream may be eliminated or transformed by several mechanisms. These include biotransformation (i.e., chemical reaction altering a substance) and biliary clearance/excretion [126]. Both mechanisms can be influenced by PA [127,128,129]. In fact, a study highlighted the ability of aerobic exercise to facilitate DDT degradation [114]. Interestingly, the authors explained that anaerobic and aerobic conditions have different biotransformation rates. A more recent study seemed to confirm the ability of PA to eliminate POPs from the human body [130]. The results demonstrated that PA can reduce benzo(a)pyrene urine levels with a sex-dependent effect. In fact, the elimination potential was more important for females than males. Secondary results showed better elimination of benzo(a)pyrene for people with a low BMI.

The excretion of POPs via urine or sweat can also contribute to the reduction of POP concentrations [131,132,133]. Nevertheless, clearance through perspiration does not impact all POPs similarly [131]. The urinary system is known to be influenced by PA, with an increase in diuresis [134]. Nonetheless, one study directly compared the effect of some POPs and an intervention including PA with kidney function, which is part of the urinary system. The results showed alteration of kidney function in relation with PFASs, but PA did not prevent this association [135]. The sweating phenomenon is increased with PA [136]. It is interesting to note that the quantities of POPs excreted may depend on the type of activity involved in sweat production [133]. More participants should be included in similar studies to confirm these results and extend the identification of the associated mechanisms. Other authors [137] mitigated the importance of POPs excretion by sweat. However, it is possible that by including all ways of excretion, the results on health may be greater. Moreover, various POPs remain to be studied when assessing sweat and urine rates of excretion.

Although POPs can be eliminated, transformed, or excreted, PA can also increase the concentration of POPs into the bloodstream. Indeed, exercise-induced lipolysis may have the potential to promote the release of POPs into the bloodstream [138] since POPs are stored in adipose tissue. The results of the previously cited study nevertheless revealed different rates of release between different PCBs. Increasing the release of POPs into the bloodstream from adipose tissues might facilitate the global reduction of POPs accumulated throughout life if combined with elimination, transformation, and excretion processes. To our knowledge, no study has addressed this issue by considering the release–elimination ratio. Yet, this is crucial information to assess the ability of PA to control the adverse effects of POPs in the context of obesity.

Furthermore, few articles have focused on POP blood concentrations during PA. Recent observations seem to indicate that the effect of PA on POP blood concentrations can vary as a function of individual characteristics (e.g., sex, age, country, exposure rate to POPs, and body composition). In addition, these recent observations also revealed that each POP category (e.g., PCBs, PBDEs, OCPs) can react differently to PA [125,139,140]. For example, in a study, OCP blood concentrations were shown to be reduced following PA, and PCB blood concentrations did not change significantly [125]. This aspect is still debated in the current literature [141,142]. Another study observed that obese individuals had significantly more plasma concentrations of various POPs than lean individuals and athletes [143]. Only DDT plasma concentrations were reported to be lower in athletes than in lean individuals.

**Figure 2:**
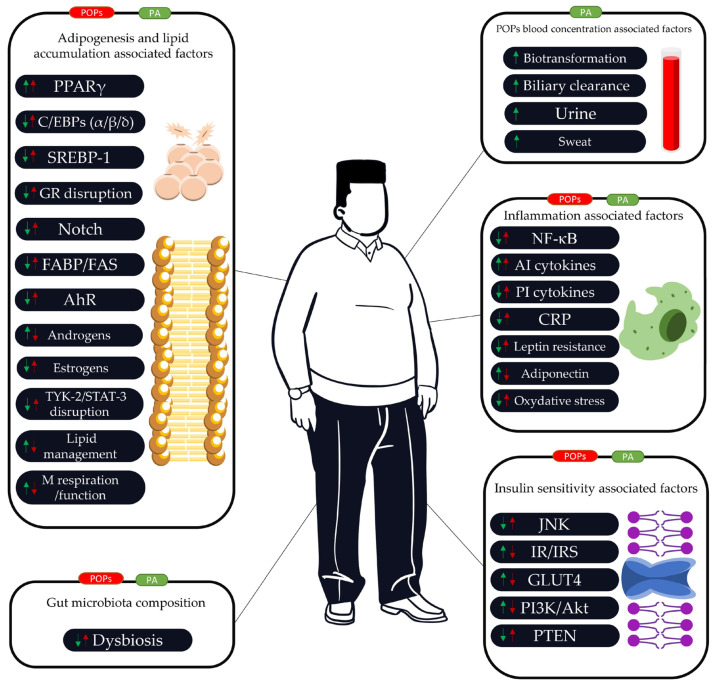
Potential mechanisms showing how physical activity (PA) may counteract the adverse effects of persistent organic pollutants (POPs) in the context of obesity. These different factors may interact with each other by direct or indirect mechanisms. With its positive impact on insulin function, lipid accumulation, adipogenesis, inflammation, and gut microbiota, PA can counteract a large variety of alterations caused by POPs. By increasing or improving physiological mechanisms such as sweat, urine, biotransformation, and biliary clearance, PA may reduce POP blood concentrations. The effects of POPs and PA can be modulated by the environment and inter-individual differences. The scheme is only an analytical summary of the isolated effects of POPs, and its validity needs to be confirmed in the case of POP cocktails or for different concentrations. Red box (global POPs’ potential negative influence), green box (global PA potential positive influence). Red arrows (potential adverse effects of POPs), green arrows (potential protective effects of PA). AhR (aryl hydrocarbon receptor), AI cytokines (anti-inflammatory cytokines), Akt (or Protein Kinase B (PKB)), AMPK (AMP-activated protein kinase), Ao-enzymes (antioxidant enzymes), C/EBPα (CCAAT enhancer-binding protein alpha), C/EBPβ (CCAAT enhancer-binding protein beta), C/EBPδ (CCAAT enhancer-binding protein delta), CRP (C-reactive protein), FABP (fatty acid-binding protein), FAS (fatty acid synthase), GLUT4 (glucose transporter type 4), IL-1β (interleukin 1 beta), IR (insulin receptor), IRS (insulin receptor substrate), JNK (Jun N-terminal kinase), lipid management (includes alterations of lipolysis, thermogenic function, triglyceride synthesis, β-oxidation, and lipid export), M respiration (mitochondrial respiration), NF-κB (nuclear factor-kappa B), PA (physical activity), PI3K (phosphoinositide 3-kinase), POPs (persistent organic pollutants), PPARγ (peroxisome proliferator-activated receptor gamma), PI cytokines (proinflammatory cytokines), PTEN (phosphatase and TENsin homolog), ROS (reactive oxygen species), SREBP-1 (sterol regulatory element-binding protein-1), TNFα (tumor necrosis factor), TYK-2/STAT-3 pathway (tyrosine kinase 2/signaling transducer and activator of transcription 3 pathway).

A schematic representation of the relations between POPs and PA in the context of obesity is presented in Figure 2.

### 3.7. Is PA an Accurate Solution to Prevent POPs’ Adverse Effects?

The present review emphasizes the performance of PA in relation to the adverse effects of POPs. However, PA could be potentially harmful in some situations if not used with caution. Third main difficulties need to be considered. The first one is the increased exposition to POPs during PA caused by environmental pollution. The second one is the increased lipolysis during PA, which facilitates POPs’ liberation into the bloodstream. The third one is the presence of POPs in physical activity equipment and associated items.

Firstly, a study reported an increased risk (×3) of exposure to a high level of POPs during PA [140]. A multifactorial approach including various respiratory parameters and mucociliary clearance (i.e., reduced during PA) can explain these results [144]. Then, practicing PA during high-pollution-level periods and/or close to POP sources would increase the inhalation of pollutants and their transport in the ventilatory tract. Another study observes the presence of a large variety of PFASs in the water of swimming pools and suggest that the presence of PFASs may be related to sunscreen, conditioners, and disinfectants [145]. Thereby, it is possible to suppose that PA should be practiced in non-polluted environments if possible. In addition, a study investigated the link between dietary intake, some POPs, and sports [146]. The results showed that different dietary habits, in comparison with the general population, could increase the risk of exposure to POPs.

Secondly, PA increases lipolysis within the adipose tissue and reduces triglycerides [147,148]. POPs being lipophilic, the degradation of lipolysis within the adipose tissue would inevitably cause POPs’ release into the bloodstream. Although increasing the release of POPs into the bloodstream from adipose tissues may facilitate the global reduction of POPs accumulated through life as previously explained, it may also cause adverse effects by facilitating the interaction of POPs with vital organs (e.g., brain, kidney, liver). This has yet to be proven. Interestingly, PFASs can have hepatotoxic effects, but PA could be an efficient strategy to prevent these effects [149]. Another study reported that weight loss can increase POP concentrations in human milk [150].

Thirdly, PA-related equipment is often fabricated with POPs, and human are regularly in contact with them. For example, a review indicated that tennis rackets, bicycles, fishing lines, climbing ropes, ski wax, and boat equipment were fabricated using PFASs [151]. Textile products are also a source of exposure [152]. Priority should be given to clothing that presents a low risk of exposure to POPs during PA. Not only can individuals be exposed to POPs by skin contact, but it is also very likely for these chemicals to affect the environment [153,154,155,156], resulting in an increased risk of exposure for humans.

This review seeks to offer insights into the potential effectiveness of PA as a strategy for mitigating the adverse effects of persistent organic pollutants within the context of obesity. A large and prolonged increase in POPs into the bloodstream of obese individuals may cause adverse effects. Such a specific situation can be observed following bariatric surgery. The next part of this review will first shortly explain the current knowledge about POPs’ blood release following bariatric surgery. Second, how PA and bariatric surgery may interact to increase or reduce POP blood concentrations will be discussed. Third, this review will speculatively attempt to question the optimization of PA strategies following bariatric surgery within the context of POPs. Understanding how obese individuals could be protected from the adverse effects of POPs’ blood release following bariatric surgery seems crucial. In this context, the use of PA could be an interesting strategy.

## 4. When Physical Activity Is Highly Recommended but Potentially Harmful: The Case of Bariatric Surgery

### 4.1. Bariatric Surgery Is Associated with an Important Increase in POP Blood Concentrations

In some cases of morbid obesity, bariatric surgery may be required. However, the massive weight loss associated with this surgical procedure may promote the release of POPs, with harmful consequences. Indeed, a recent review emphasized that an individual’s weight loss is accompanied by an increase in POP concentrations of 2–4% per kilogram of weight loss [11]. A recent study also evidenced that blood concentrations of POPs may double in the year following bariatric surgery [157]. However, the magnitude of the concentration increase may vary depending on the molecule considered. It is nevertheless important to note that in some cases, the increases could exceed the current recommendations [158]. Interestingly, it is also observed that certain characteristics, such as the type of POP released, may vary among populations following bariatric surgery. This makes each person a unique case to be treated. Furthermore, the increase in POP concentrations following bariatric surgery has been shown to be potentially associated with hormonal changes in humans [159]. Finally, the ability of POPs to spread to other organs (e.g., like the brain) via the bloodstream should be considered [160].

### 4.2. How to Implement PA Programs to Protect against POPs’ Release

**Figure 3:**
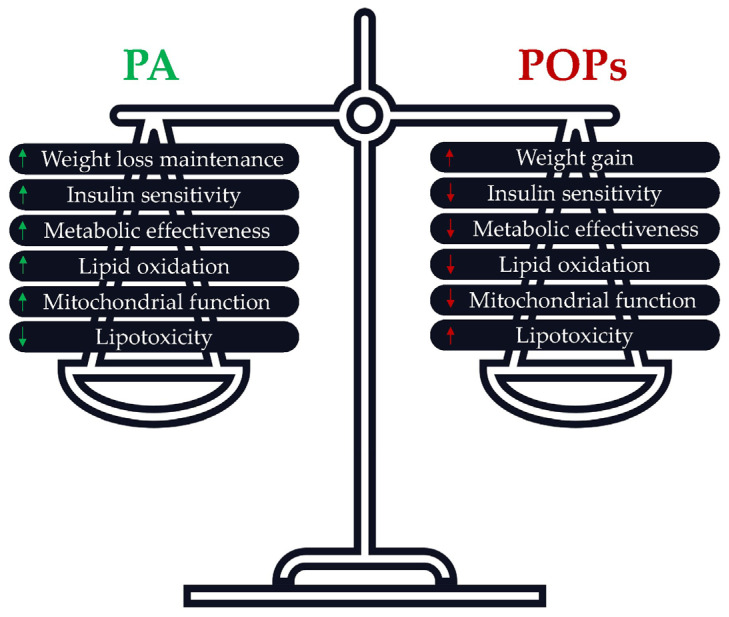
Potential effects of PA and POPs post-bariatric surgery. Green arrows (potential beneficial effects of PA post bariatric surgery), Red arrows (potential adverse effects of POPs post bariatric surgery).

PA is highly recommended to improve patients’ health, wellbeing, and quality of life after bariatric surgery. PA also favors weight loss, preserves muscle quality, and improves cardiorespiratory capacities [161]. However, after bariatric surgery, it remains to be determined if PA would have beneficial or adverse effects on POPs’ release. This reasoning is based on the rationale linking PA, lipolysis, and POPs’ release into the bloodstream. As already mentioned, it is suggested that by promoting lipolysis, PA could increase POP plasma concentrations beyond current sanitary regulation levels. Alternatively, one may suggest that PA could promote POPs’ elimination. This review identified the potential protective effects of PA against POPs’ adverse effects. It is therefore important to assess whether PA can also counteract the adverse effects of POPs’ release following bariatric surgery. An overview of the potential beneficial effects of PA post-bariatric surgery [162] and of the potential adverse effects of POPs is given in Figure 3.

**Figure 4:**
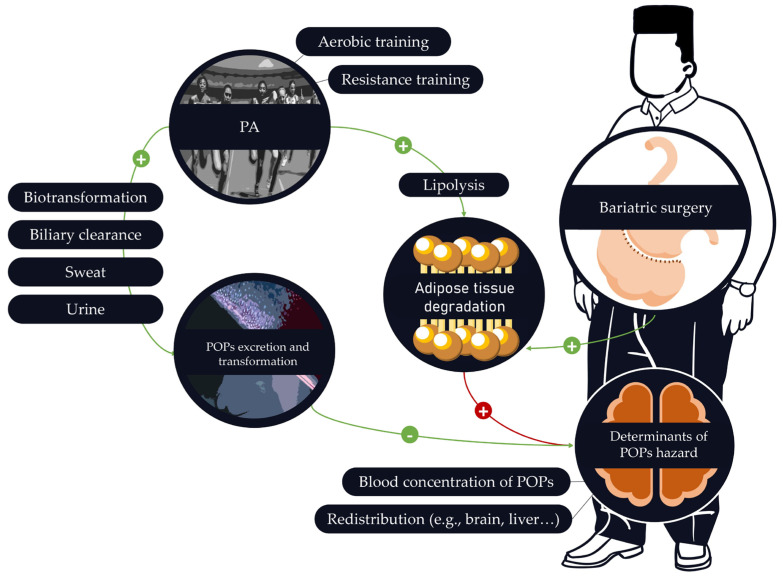
The possible relationships between bariatric surgery, persistent organic pollutants (POPs), and physical activity (PA). Physical activity as well as bariatric surgery can promote POPs’ adipose tissue degradation, resulting in an increase in several determinants of POPs’ hazards. The balance between POPs’ release following adipose tissue degradation and POPs’ excretion/transformation may depend on the physical activity modality (aerobic vs. resistance training). Minus and plus signs in green (potential beneficial effects post bariatric surgery), Plus sign in red (potential adverse effects post bariatric surgery).

More studies are needed to confirm these results and explore other previously cited parameters in the context of bariatric surgery. Opposite effects may be modulated by the type of PA involved in the rehabilitation process. After bariatric surgery, aerobic training and resistance training can be combined. Resistance training can be implemented for its beneficial effects on muscle mass and strength and aerobic training for its beneficial effects on cardiovascular, visceral adiposity, and respiratory capacities [163,164,165,166]. Each type of PA seems to have specific benefits to offer, but aerobic training promoting fat loss could induce a greater release of POPs into the bloodstream (Figure 4). Alternatively, resistance training would not expose individuals to these adverse effects.

**Figure 5:**
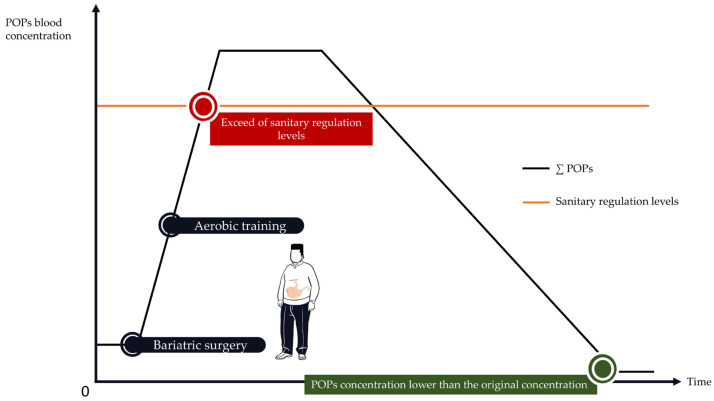
The hypothetical adverse consequences of using aerobic training shortly after bariatric surgery. Aerobic training used shortly after bariatric surgery may increase lipolysis and POPs’ release into the blood.

In this context, it could be suggested to periodize the different types of PA (i.e., aerobic training and resistance training) during the readaptation period to protect the individual against POPs’ release into the bloodstream. For instance, given that POP levels increase during the first months following bariatric surgery, resistance training should be planned during this initial period to limit POPs’ release into the blood through reduced lipolysis. Aerobic training should be periodized after this initial phase, when POPs’ release is reduced or after the spike. However, this is purely speculative. Studies are needed to confirm or refute this proposal. Alternatively, programming aerobic exercise shortly after surgery may contribute to exceeding sanitary regulation levels. It may also cause a harmful release of obesogenic POP mixtures into the bloodstream (Figure 5).

**Figure 6:**
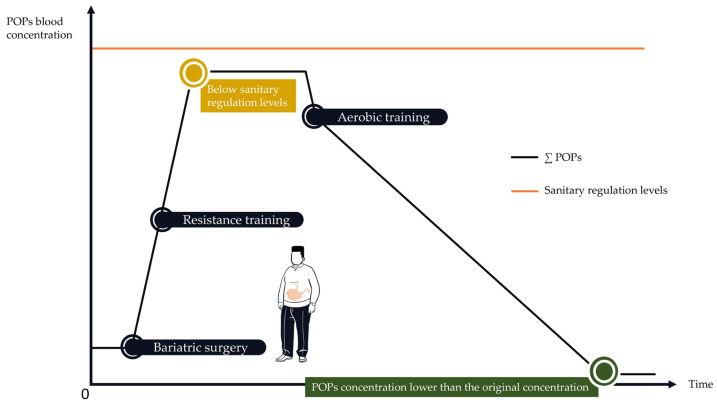
Hypothetical utilization of resistance training and aerobic exercise following bariatric surgery to protect the individual from POPs’ blood release.

Early periodization of resistance training could prevent overcoming POP sanitary regulation levels. Resistance training in combination with bariatric surgery may produce less POPs’ release into the bloodstream than aerobic training after bariatric surgery. When POP blood concentrations start to drop, aerobic training could be implemented (Figure 6). However, POPs’ dangerousness may not follow a linear dose–response model, in which case, increased POP blood concentrations may not be the ideal biomarker to determine which type of PA should be used and when. Therefore, future studies should assess the relationship between PA modality, POPs’ mobilization rate, lipolysis-induced POPs’ blood release rate, and POPs’ concentration dangerousness. The type, intensity, and frequency of PA may also be responsible for inter-individual variations. It is possible to assume that an individual practicing PA regularly will have greater benefits than an individual with an irregular practice of PA. More PA sessions could lead to greater chances of excreting POPs from the body. It is also possible that increasing the exercise intensity would result in greater benefits. An individual with high-intensity PA may therefore be better protected from the adverse effects of POPs than an individual with low-intensity PA through increased sweat production. Inter-individual variability could also be explained by the different ways of practicing PA. It could be questioned whether an individual practicing interval aerobic training will have similar POPs’ mobilization as an individual practicing continuous aerobic training. As explained above, the conditions in which PA is practiced can influence exposure to POPs. An individual practicing in a polluted environment could therefore benefit less than an individual practicing in an unpolluted environment. Finally, physiological differences (e.g., altered bile system, altered sweat production, altered inflammation system) could also explain inter-individual differences.

## 5. Conclusions

POPs are increasingly known for their ability to promote the development of obesity. Among the main reasons, there is a possible detrimental effect of POPs on adipogenesis, lipid accumulation, insulin resistance, inflammation, and gut dysbiosis. All these factors can be influenced by PA, and our analysis of the literature suggests that PA could potentially be an effective intervention to counteract most of the adverse effects of identified POPs. However, only a few studies have directly assessed the effects of PA on these adverse effects in the context of exposure to POPs. PA also appears to be associated with the direct mobilization of POPs, but the ins and outs of this mobilization are less known. For example, it is unclear whether the excretion of POPs via sweat during PA is sufficiently important to limit their accumulation into the bloodstream consecutive to lipolysis. Future studies should address this question.

It is possible to assume that the “POPs adipose tissue profile”, including diversity and quantity, is the key factor to understand the development of obesity in some individuals. To give an example, it is logical to assume that a person with an “obesogenic POPs profile” (i.e., quantity and variety favoring the development of obesity) is more at risk to develop obesity than a person with a “non-obesogenic POPs profile” (i.e., quantity and variety less at risk for obesity but which may present other health risks). To our knowledge, no study has established a global profile for a person living with obesity. Although the cost may be very expensive, future studies should aim to assess these profiles and to establish the effect of PA on all these profiles.

## Data Availability

This article is a narrative review. No data were generated.

## Supplementary Materials

The following supporting information can be downloaded at: https://www.mdpi.com/article/10.3390/ijms25020883/s1.

## Abbreviations

ACSM	American College of Sports Medicine
AhR	Aryl hydrocarbon Receptor
Akt	or Protein Kinase B (PKB)
AMPK	AMP-Activated Protein Kinase
APA	Adapted Physical Activity
ATM/NEMO pathway	Ataxia Telangiectasia Mutated/NF-κB Essential Modifier pathway
BaP	Benzo(a)Pyrene
C/EBPα	CCAAT Enhancer-Binding Protein alpha
C/EBPβ	CCAAT Enhancer-Binding Protein beta
C/EBPδ	CCAAT Enhancer-Binding Protein delta
CAT	Chloramphenicol AcetylTransferase
CCAAT	Cytosin-Cytosin-Adenosin-Adenosin-Thymidin
CCL2	Macrophage Chemoattractant Protein-1 (MCP-1)
CCL3	Macrophage Inflammatory Protein-1 alpha (MIP-1α)
CCL4	Macrophage Inflammatory Protein-1 beta (MIP-1β)
CRP	C-Reactive Protein
DDT	DichloroDiphenylTrichloroethane
EASO	European Asylum Support Office
eNOS	Endothelial Nitric Oxide Synthase
FABP	Fatty Acid-Binding Protein
FAS	Fatty Acid Synthase
FFAs	Free Fatty Acids
GLUT4	Glucose Transporter type 4
GR	Glucocorticoid Receptor
GSH-Px	Glutathion Peroxydase
IFNγ	Interferon-gamma
IκB	Inhibitor κB
IL-12p70	Interleukin 12p70
IL-17A	Interleukin 17A
IL-1Ra	Interleukin-1Ra
IL-1β	Interleukin 1beta
IL-2	Interleukin 2
IL-5	Interleukin 5
IL-6	Interleukin 6
IL-8	Interleukin 8
INSERM	Institut National de la Santé et de la Recherche Médicale
IRS	Insulin Receptor Substrate
IVM	Integrated Vector Management
JNK	Jun N-terminal Kinase
LPL	Lipoprotein Lipase
MDA	Malondialdehyde
NADPH oxidase	Nicotinamide Adenine Dinucleotide Phosphate oxidase
NF-κB	Nuclear Factor-Kappa B
OCPs	OrganoChlorine Pesticides
PA	Physical Activity
PBDEs	PolyBromoDiphénylEthers
PCBs	PolyChlorinated Biphenyls
PGC1α	Peroxisome Proliferator-Activated Receptor Gamma Coactivator 1-alpha
PI3K	Phosphoinositide 3-Kinase
POPs	Persistent Organic Pollutants
PPARγ	Peroxisome Proliferator-Activated Receptor gamma
PTP1B	Protein Tyrosine Phosphatase 1B
PTEN	Phosphatase and TENsin homolog
ROS	Reactive Oxygen Species
SHBG	Sex Hormone Binding Globulin
SOCS3	Supressor Of Cytokine Signaling 3
SOD	SuperOxyde Dismutase
SREBP-1	Sterol Regulatory Element-Binding Protein-1
TNFα	Tumor Necrosis Factor alpha
TYK-2/STAT-3	Tyrosine Kinase-2/Signal Transducer and Activator of Transcription 3
VAT	Visceral Adipose Tissue
