# Supplementary material for: Is Physical Activity an Efficient Strategy to Control the Adverse Effects of Persistent Organic Pollutants in the Context of Obesity? A Narrative Review
Source: Int J Mol Sci. 2024 Jan 10;25(2):883. doi: 10.3390/ijms25020883 (PMC10815489; doi:10.3390/ijms25020883)
Supplement: Supplementary file 1 [file ijms-25-00883-s001.zip › ijms-2744277-supplementary.pdf]

Methodology used to select the articles included in the review

This narrative review is based on scientific literature published before July 2023. The literature has been explored between October 1^{sts} 2022 and August 1st of 2023. Only articles directly assessing the interactions between POPs and PA or related terms were selected through a specific process: (persistent organic pollutants OR dioxin OR aldrin OR chlordane OR chlordecone OR decabromodiphenyl ether OR dicofol OR dieldrin OR endrin OR heptachlor OR hexabromobiphenyl OR hexabromocyclododecane OR hexabromodiphenyl ether OR heptabromodiphenyl ether OR hexachlorobenzene OR hexachlorobutadiene OR alpha hexachlorocyclohexane OR beta hexachlorocyclohexane OR Lindane OR mirex OR pentachlorobenzene OR pentachlorobenzene OR pentachlorophenol OR polychlorinated biphenyl OR polychlorinated naphthalene OR perfluorooctanoic acid OR perfluorohexane sulfonic acid OR short-chain chlorinated paraffin OR technical endosulfan OR tetrabromodiphenyl ether OR pentabromodiphenyl ether OR toxaphene OR dichlorodiphenyltrichloroethane OR perfluorooctane sulfonic acid OR polyfluoroalkyl substances OR PFAS OR perfluorooctane sulfonyl fluoride OR polychlorinated dibenzo-p-dioxin OR polychlorinated dibenzofurans OR organochlorine pesticides OR PFAS OR polyfluoroalkyl substances, OR perfluoroalkyl substances) AND (physical activity OR exercise OR training OR athlete OR sport). Articles related to POPs combined with PA and associated terms were found on PubMed using the specific request. Exclusion criteria included non-English articles, methodological articles, reviews, and articles without combination of POPs related terms with PA activity related terms. Incomplete articles were also excluded. Letters were included for their relevance. The research process and the articles considered are summarized in the flowchart below (Figure ~~5~~S1). Other articles (e.g., related to the POPs adverse effects without PA) were selected because of their pertinence but not with a specific methodology.

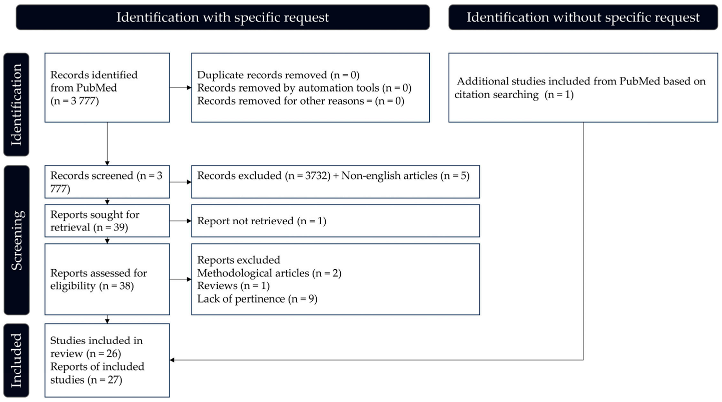

Figure ~~5~~S1: Flowchart: Representation of the methodology used during articles selection.
